# Concentrations of perfluoroalkyl and polyfluoroalkyl substances and blood glucose in pregnant women

**DOI:** 10.1186/s12940-020-00640-8

**Published:** 2020-08-17

**Authors:** Yanfeng Ren, Longmei Jin, Fen Yang, Hong Liang, Zhaofeng Zhang, Jing Du, Xiuxia Song, Maohua Miao, Wei Yuan

**Affiliations:** 1 Department of Health Statistics, School of Public Health, Weifang Medical University, Weifang, Shandong China; 2 Minhang District Maternal and Child Health Hospital, Shanghai, China; 3 Department of Global Public Health, Karolinska Institute, Stockholm, Sweden; 4 NHC Key Lab of Reproduction Regulation (Shanghai Institute of Planned Parenthood Research), Fudan University, Shanghai, China

**Keywords:** Perfluoroalkyl and polyfluoroalkyl substances, Plasma glucose, Cohort study, Pregnancy

## Abstract

**Background:**

Evidence on the association between exposure to perfluoroalkyl and polyfluoroalkyl substances (PFASs) and blood glucose concentrations in pregnant women is inconsistent. This study aimed to examine the association between PFAS exposure and the concentrations of fasting plasma glucose (FPG) and one-hour plasma glucose (1 h-PG) after a 50-g oral glucose tolerance test in pregnant women.

**Methods:**

The study was based on the Shanghai-Minhang Birth Cohort, in which 1292 pregnant women were recruited. Among them, 981 women provided blood samples (at 12–16 gestational weeks) for PFAS measurement. FPG data collected from 856 women at 12–20 GW and 1 h-PG data collected from 705 women at 20–28 GW were obtained through medical records from the routine prenatal care system. High FPG or 1 h-PG was defined as ≥90th percentile of FPG or 1 h-PG. The analysis of eight PFASs was conducted in this study: perfluorohexane sulfonate (PFHxS), perfluorooctane sulfonate (PFOS), perfluorooctanoic acid (PFOA), perfluorononanoic acid (PFNA), perfluorodecanoic acid (PFDA), perfluoroundecanoic acid (PFUdA), perfluorododecanoic acid (PFDoA), and perfluorotridecanoic acid (PFTrDA). The odds ratios (ORs) and associated 95% confidence intervals (CIs) were estimated to determine the associations of each PFAS compound with high FPG and 1 h-PG from a logistic regression model.

**Results:**

After adjustment for potential confounders, most PFASs were positively associated with high 1 h-PG concentrations. The OR for high 1 h-PG concentrations was 1.87 (95% CI: 1.15–3.05) with a one log unit increase of PFOS; similar associations were observed for PFNA (OR: 2.15, 95% CI: 1.24–3.74), PFDA (OR: 1.61, 95% CI: 1.10–2.44), PFUdA (OR: 1.71, 95% CI: 1.12–2.62), and PFDoA (OR: 1.34, 95% CI: 1.00–1.81). When the PFAS concentrations were categorized into three groups by tertiles, the highest tertiles of PFOS, PFOA, PFNA, PFDA, PFDoA, and PFTrDA had a statistically significant increase in the risk of high 1 h-PG concentrations compared with the lowest tertiles. No statistically significant association was observed between PFAS exposure and high FPG.

**Conclusion:**

PFAS exposure was associated with an increased risk of high 1 h-PG among pregnant women, but no such association was observed for FPG.

## Introduction

Perfluoroalkyl and polyfluoroalkyl substances (PFASs), a group of man-made chemicals with water-, stain-, and grease-resistant properties, are used in a wide range of consumer products, including fast food packaging, stain-resistant carpets, windshield washing fluid, fire-fighting foam, insecticides, and paints [1]. Humans are widely exposed to PFASs through the ingestion of contaminated drinking water and food, as well as the inhalation of contaminated indoor air and dust [2]. Some PFASs have been shown to bio-accumulate in organisms [3]. The mean half-lives of PFASs in adult humans vary from 2.3 to 8.5 years [4, 5]. The most commonly studied PFASs, including perfluorohexane sulfonate (PFHxS), perfluorooctane sulfonate (PFOS), perfluorooctanoate (PFOA), and perfluorononanoate (PFNA), are detected in the majority of human serum samples [6].

Animal studies have shown that PFAS exposure is associated with a wide range of adverse health effects, including the disruption of endocrine hormones, such as testosterone, estrogen, and thyroid hormones [7, 8], alterations in serum lipid levels [9], impaired glucose metabolism and insulin hypersensitivity [10], and immune system disturbance [1, 9]. Human studies have also suggested the adverse effects of PFASs on the immune system [11], carcinogenesis [12], pregnancy-induced hypertension, arterial atherosclerosis [13, 14], and glucose metabolism [15–19].

Although epidemiological studies have suggested that PFASs are associated with impaired glucose tolerance and homeostasis, insulin resistance, beta-cell dysfunction, and a higher risk of diabetes [15–19], the associations observed in the general population cannot be generalized to metabolically vulnerable pregnant women owing to their special, insulin-resistant state during pregnancy. The current evidence on the effects of PFASs on glucose metabolism in pregnant women is limited and inconclusive. In the Odense Child Cohort study, PFHxS and PFNA concentrations were associated with impaired glycemic status in pregnant women and may therefore enhance the risk of developing gestational diabetes mellitus (GDM) [20]. In another prospective study of 258 women, higher pre-pregnancy PFOA concentrations were associated with an increased risk of GDM, but the associations for six other PFASs were not statistically significant [21]. In contrast, Valvi et al. found no associations between PFOA, PFOS, PFHxS, PFNA, or perfluorodecanoic acid (PFDA) concentrations and the risk of GDM in pregnant women [22].

In the present study, we sought to evaluate the associations between PFAS exposure and fasting plasma glucose (FPG) and 1-h plasma glucose concentrations (1 h-PG) measured after a 50-g oral glucose tolerance test (OGTT) in pregnant women by using data from the Shanghai-Minhang Birth Cohort Study (S-MBCS).

## Methods

### Study participants

All study participants were recruited from the S-MBCS between April 2012 and December 2012. Pregnant women attending their first routine antenatal care at the Maternal and Child Health Hospital of Minhang district in Shanghai were consecutively recruited if: 1) they were at 12–16 gestational weeks (GW) of pregnancy; 2) they were registered residents of Shanghai; 3) they had no history of chronic disease of the liver, kidney, or other organs; 4) they planned to give birth in the study hospital; and 5) they were willing to participate in specified interviews during pregnancy and after delivery. Among 1670 pregnant women who were invited, 1292 pregnant women were recruited, corresponding to a response rate of 77.4%.

### Exposure assessment and quality control

Blood samples for PFASs measure were collected at recruitment, and plasma samples were separated and stored at − 80 °C, before they were transported to the Center for Disease Control and Prevention in Hubei Province for the assay of PFAS.

High-performance liquid chromatography coupled with tandem mass spectrometry (Agilent Technologies Inc., USA) was used for the quantitative measurement of PFASs. The information on sample collection, separation, reservation, transportation, quantification, limit of detection (LOD), and quality control has been detailed previously [6]. Among the 11 PFASs measured in our study, eight PFASs with detection rates above 90%, including PFHxS, PFOS, PFOA, PFNA, PFDA, perfluorododecanoic acid (PFDoA), perfluoroundecanoic acid (PFUdA), and perfluorotridecanoic acid (PFTrDA), were included in the final analyses.

An internal standard approach was used to aid quantification. Milli-Q water was used to perform procedural blank analysis for each batch of samples. The concentrations of each detected congener should be more than three times of that in the procedural blank, and were corrected by subtracting the procedural blank concentration in the present study. LOD was defined as the concentration with a signal-to-noise ratio equal to or greater than 3. All the recoveries ranged from 71.5–112.3%. A five point calibration curve was drawn and each precursor ranged from 0.02–20.00 ng/mL. Calibration curves presented a linear pattern over the concentration range of the precursor.

### Glucose and covariate measurement

The information on plasma glucose concentrations in pregnant women was collected from the medical records of the prenatal care system, and included results for FPG and 1 h-PG. In the study hospital within the study period, pregnant women were asked to provide blood after overnight fasting for FPG testing at their earliest conveniences, generally within 1 week after their first antenatal care. It was suggested that pregnant women underwent a 1 h 50-g OGTT at 20 GW in order to screen for gestational diabetes, if they were considered to have a high risk of GDM(*n* = 37), i.e., FPG ≥ 6.1 mmol/L (110 mg/dL) [23], or overweight and obese(i.e., BMI ≥ 25 kg/m^2^) [24]; otherwise, it was suggested that the examination of 1 h-PG was performed between 24 and 28 GW. The 50-g OGTT was performed after an overnight fasting, also. The distribution of gestational weeks in which the FPG and 1 h-PG examination was performed is shown in Supplemental Table S1. Information on whether the women had been diagnosed with GDM was extracted through medical records at birth.

A structured questionnaire was used by trained interviewers to collect information on the covariates. The women were asked about age, per capita household income, education level, passive smoking, height, pre-pregnancy weight, parity, history of abortion and stillbirth, pregnancy complications, etc. Pre-pregnancy BMI (kg/m^2^) was calculated as body weight divided by body height squared.

### Statistical analysis

**Fig. 1:**
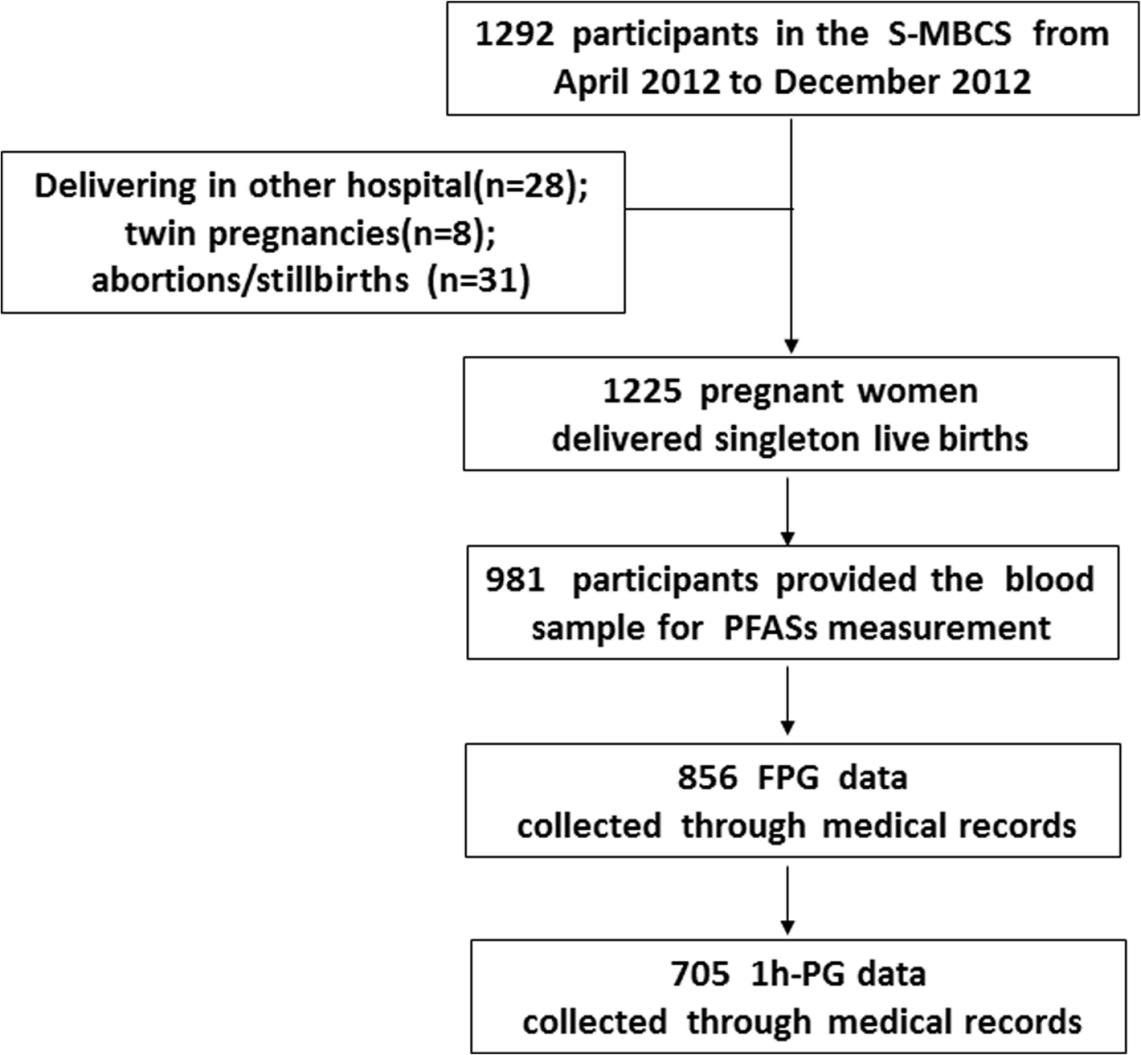
Study population of the present study from S-MBCS. * FPG, fasting plasma glucose; 1 h-PG, 1 h-plasma glucose after a 50-g oral glucose tolerance test; S-MBCS, Shanghai-Minhang Birth Cohort Study

Among the 1292 pregnant women recruited, 1225 women delivered singleton live births, and 981 women provided blood samples at enrollment for PFAS measurement. FPG concentrations measured at 12–20 GWs were obtained for 856 women, and 1 h-PG concentrations measured at 20–28 GWs were obtained for 705 pregnant women. Pregnant women who had data on PFASs and FPG concentrations were included in this study (Fig. 1). We first described and compared the demographic characteristics of the included and excluded pregnant women. The means and standard deviations (SD) were used to describe the distributions of FPG and 1 h-PG according to the demographic characteristics of the included pregnant women. A logistic regression model was used to examine the association between PFAS exposure and plasma glucose, with the 90th percentiles of FPG (4.6 mmol/L, i.e., 83 mg/dL) and 1 h-PG (8.3 mmol/L, i.e., 149 mg/dL) used as the cutoff value. Natural logarithm (ln)-transformed PFAS concentrations were first included in logistic regression models, and those with concentrations below the LOD were assigned a value of LOD/√2. PFAS concentrations were also categorized into three groups by tertiles (T1: lowest tertile; T2: middle tertile; and T3: highest tertile) and included in the logistic regression models with the lowest tertile as the reference group. Odds ratios (ORs) and associated 95% confidence intervals (CIs) were estimated for the association between each PFAS and high FPG/1 h-PG (i.e., ≥90th of FPG concentration or ≥ 90th of 1 h-PG concentration). Based on tertiles, the concentrations were transformed to ordinal data and assigned to all persons to calculate p-trend values. In addition, multiple linear regressions were used to analyze the association between PFASs and continuous plasma glucose concentrations.

Potential confounders were identified a prior according to the previous literature. Age of pregnant women, education, economic income, pre-pregnancy BMI, passive smoking, parity, history of abortion and stillbirth and pregnancy complications, including bleeding, thyroid disease and pregnancy-induced hypertension [20, 21, 25] were identified, and a directed acyclic graphs (Supplemental Figure S1) was used to evaluate the appropriation of covariates. We did not adjust for alcohol consumption(*n* = 6) in the final models because of the low prevalence. The statistical assumptions of logistic regressions were evaluated and met, including linear relationship of independent variables with logit(p), outliers, and colinearity of independent variables.

Several sensitivity analyses were performed to test the robustness of the primary results:1) Considering the potential effect of pre-pregnancy BMI on GDM [20] and the variation in PFAS concentrations across BMI, we repeated the analysis in women with a pre-pregnancy BMI of < 25 kg/m^2^ to eliminate the confounding effect of BMI. 2) To test the generalizability of the results, we repeated the analyses in pregnant women without GDM. 3) To examine whether the associations of PFASs with FPG/1 h-PG were time-dependent, we performed subgroup analyses for different spans of GW at glucose measurement (for FPG, at 12–14 GWs and 15–20 GWs; for 1 h-PG, at 20–23 GWs and 24–28 GWs). Statistical Analysis System (SAS) software version 9.3 (SAS Institute, Inc., Cary, NC, USA) was used for statistical analysis. *P* values of < 0.05 were considered statistically significant.

## Results

Table 1 presents the characteristics of the included pregnant women are compared with those of the excluded women in the study. The majority of women included in the present analyses were nulliparous (86.1%), 25–35 years of age (81.43%), with a BMI between 18.5–24.9 kg/m^2^ (74.17%), with a household income per capita of > 4000 CNY/month, well educated (76.46% college-level education or above), without pregnancy complication (93.8%), without history of abortion and stillbirth (62.6%). Approximately 40% of women were exposed to passive smoking during pregnancy. The distributions of these demographic characteristics were not significantly different between the included and excluded women except parity.

**Table 1:** Characteristics of the included and excluded pregnant women

Characteristics	Included (*N* = 856) N (%) / Mean ± SD	Excluded (*N* = 369) N (%) / Mean ± SD	*P*-value of Student’s t-test or Chi-square test
Maternal age at enrollment (years)			
Mean ± SD	27.8 ± 3.3	27.8 ± 3.4	0.784
< 25	128 (15.0)	56 (15.2)	0.454
25–35	697 (81.4)	294 (79.7)	
≥ 35	31 (3.6)	19 (5.1)	
Pre-pregnancy BMI (kg/m^2^)			
Mean ± SD	20.4 ± 2.4	20.5 ± 2.4	0.732
< 18.5	176 (20.9)	68 (18.7)	0.666
18.5–24.9	627 (74.6)	279 (76.6)	
≥ 25	37 (4.4)	17 (4.7)	
Maternal education			
Below high school	76 (8.9)	43 (11.7)	0.314
High School	125 (14.6)	50 (13.5)	
College or above	653 (76.5)	276 (74.8)	
Per capita household income (CNY)			
< 4000	176 (21.0)	57 (16.2)	0.151
4000–8000	495 (59.1)	224 (63.8)	
> 8000	167 (19.9)	70 (19.9)	
Passive smoking			
Yes	295 (40.4)	109 (43.9)	0.320
No	436 (59.6)	139 (56.1)	
Pregnancy complication			
Yes	53 (6.19)	22 (6.0)	0.878
No	803 (93.8)	347 (90.0)	
History of abortion and stillbirth			
Yes	320 (37.4)	147 (39.8)	0.271
No	536 (62.6)	222 (60.2)	
Parity			
0	732 (86.1)	296 (80.8)	0.020
≥ 1	118 (13.9)	70 (19.1)	

Table 2 presents PFHxS, PFOS, PFOA, PFNA, and PFDA were detected in all maternal plasma samples, while PFUdA, PFDoA and PFTrDA were detected in about 90% samples. PFOA and PFOS had the highest concentrations (PFOA: *GM* = 20.2 ng/mL; PFOS: *GM* = 10.8 ng/mL), followed by PFHxS (*GM* = 2.7 ng/mL), PFDA (2.2 ng/mL), PFNA (1.8 ng/mL), and PFUdA (1.6 ng/mL), while PFDoA and PFTrDA had the lowest concentrations.

**Table 2:** PFASs concentrations (ng/mL) of the included pregnant women (*N* = 856)

PFAS	LOD	>LOD (N %)	GM (GSD)	Percentiles				
				5th	25th	50th	75th	95th
PFHxS	0.015	856 (100)	2.7 (1.5)	1.3	2.1	2.8	3.6	5.6
PFOS	0.02	856 (100)	10.7 (1.7)	4.2	7.6	10.7	15.7	25.6
PFOA	0.01	856 (100)	19.9 (1.6)	9.3	15.5	20.2	27.0	38.8
PFNA	0.02	856 (100)	1.8 (1.6)	0.8	1.3	1.8	2.5	4.0
PFDA	0.01	856 (100)	2.0 (1.9)	0.7	1.3	2.0	3.2	6.2
PFUdA	0.01	855 (99.9)	1.6 (2.0)	0.5	1.0	1.5	2.54	4.5
PFDoA	0.015	776 (90.6)	0.1 (2.2)	LOD	0.1	0.1	0.2	0.4
PFTrDA	0.02	745 (87.0)	0.1 (2.1)	LOD	0.1	0.1	0.2	0.4

*Note*: *LOD* limit of detection, *GM* geometric mean, *GSD* geometric standard deviation

Table 3 presents the concentrations of FPG and 1 h-PG according to the demographic characteristics of the subjects. The mean (SD) FPG and 1 h-PG concentrations were 4.04 (0.45) mmol/L (i.e., 72.7(8.1) mg/dL) and 6.46 (1.37) mmol/L (i.e., 116.3(24.7) mg/dL), respectively. The concentrations of FPG and 1 h-PG were comparable across pregnant women with different BMI, household income, passive smoking status, pregnancy complication and history of abortion and stillbirth. The concentration of 1 h-PG was higher in pregnant women who were older or had higher education levels, but not in those with FPG. The concentration of FPG was lower in nulliparous pregnant women, but not in those with 1 h-PG.

**Table 3:** The distribution of FPG and 1 h-PG (mmol/L) according to participant’s demographic characteristics

Characteristics	FPG(*N* = 856)		1 h-PG(*N* = 705)	
	Mean ± SD	*P*-value	Mean ± SD	*P*-value
Total	4.04 ± 0.45		6.46 ± 1.37	
Maternal age at enrollment (years)		0.675		< 0.001
< 25	4.01 ± 0.39		5.99 ± 1.28	
25–35	4.04 ± 0.45		6.54 ± 1.35*	
≥ 35	4.08 ± 0.49		6.73 ± 1.70*	
Pre-pregnancy BMI (kg/m^2^)		0.854		0.097
< 18.5	4.06 ± 0.47		6.25 ± 1.38	
18.5–24.9	4.04 ± 0.44		6.52 ± 1.36	
≥ 25	4.09 ± 0.45		6.65 ± 1.43	
Maternal education		0.077		0.032
Below high school	4.15 ± 0.44		6.05 ± 1.55	
High School	4.05 ± 0.46		6.41 ± 1.54	
College or above	4.02 ± 0.44		6.52 ± 1.30*	
Per capita household income (CNY)		0.341		0.702
< 4000	4.05 ± 0.39		6.46 ± 1.33	
4000–8000	4.05 ± 0.46		6.49 ± 1.37	
> 8000	3.99 ± 0.47		6.37 ± 1.39	
Passive smoking		0.512		0.803
Yes	4.07 ± 0.47		6.44 ± 1.40	
No	4.05 ± 0.43		6.47 ± 1.38	
Pregnancy complication		0.725		0.146
Yes	4.28 ± 0.43		6.76 ± 1.54	
No	4.04 ± 0.45		6.45 ± 1.39	
History of abortion and stillbirth		0.156		0.426
Yes	4.25 ± 0.43		6.52 ± 1.5	
No	4.07 + 0.43		6.44 + 1.33	
Parity		0.003		0.059
0	4.07 ± 0.48		6.43 ± 1.36	
≥ 1	4.15 ± 0.43		6.72 ± 1.61	

*FPG* fasting plasma glucose, *1 h-PG* 1 h-plasma glucose after a 50-g oral glucose tolerance test

*, *p* < 0.05, compared with the first group

Table 4 presents that higher concentrations of PFOS, PFOA, PFNA, PFDA, PFDoA, and PFTrDA were associated with an increased risk of high FPG; however, the associations were not statistically significant (AOR_PFOS_ = 1.28, 95% CI: 0.85–1.93; AOR_PFOA_ = 1.31, 95% CI: 0.77–2.22; AOR_PFNA_ = 1.55, 95% CI: 0.98–2.46; AOR_PFDA_ = 1.24, 95% CI: 0.87–1.76; AOR_PFDoA_ = 1.09, 95%CI: 0.87–1.37; AOR_PFTrDA_ = 1.12, 95% CI: 0.89–1.40). Higher concentrations of PFASs were associated with an increased risk of high 1 h-PG, except for PFHxS, and the associations with PFOS, PFNA, PFDA, PFUdA, and PFDoA were statistically significant after adjustment for potential confounders (AOR_PFOS_ = 1.87, 95% CI: 1.15–3.05; AOR_PFNA_ = 2.15, 95% CI: 1.24–3.74; AOR_PFDA_ = 1.61, 95% CI: 1.10–2.44; AOR_PFUdA_ = 1.71, 95% CI: 1.12–2.62; AOR_PFDoA_ = 1.34, 95% CI: 1.00–1.81). In addition, multiple linear regressions were also used to analyze the association between PFASs and plasma glucose. Similar results were found in multiple linear regression as in logistic regression model, although the association of PFDoA with 1 h-PG is not statistically significant (Supplemental Table S2).

**Table 4:** Association between PFAS concentrations (ln-transformed) and high FPG and 1 h-PG in pregnant women

In-PFAS (ng/ml)	FPG(*N* = 856)		1 h-PG(*N* = 705)	
	COR (95% CI)	AOR (95% CI)	COR (95% CI)	AOR (95% CI)
PFHxS	1.00 (0.61, 1.62)	0.89 (0.51, 1.55)	0.92 (0.52, 1.63)	0.92 (0.45, 1.86)
PFOS	1.28 (0.88, 1.85)	1.28 (0.85, 1.93)	1.77 (1.15, 2.72)	1.87 (1.15, 3.05)
PFOA	1.39 (0.86, 2.26)	1.31 (0.77, 2.22)	1.15 (0.67, 1.92)	1.40 (0.75, 2.59)
PFNA	1.54 (1.02, 2.34)	1.55 (0.98, 2.46)	1.74 (1.08, 2.78)	2.15 (1.24, 3.74)
PFDA	1.25 (0.91, 1.71)	1.24 (0.87, 1.76)	1.52 (1.06, 2.17)	1.61 (1.10, 2.44)
PFUdA	0.99 (0.73, 1.34)	0.98 (0.71, 1.35)	1.48 (1.04, 2.12)	1.71 (1.12, 2.62)
PFDoA	1.13 (0.92, 1.39)	1.09 (0.87, 1.37)	1.28 (1.00, 1.65)	1.34 (1.00, 1.81)
PFTrDA	1.04 (0.85, 1.28)	1.12 (0.89, 1.40)	1.12 (0.88, 1.41)	1.13 (0.87, 1.49)

*COR* crude odds ratio, *AOR* adjusted odds ratio, *CI* confidence interval, *FPG* fasting plasma glucose, *1 h-PG* 1 h-plasma glucose after a 50-g oral glucose tolerance test

Models were adjusted for maternal age at enrollment (years), pre-pregnancy BMI (kg/m^2^), per capita household income, education level, passive smoking, pregnancy complication, history of abortion and stillbirth, and parity

**Fig. 2:**
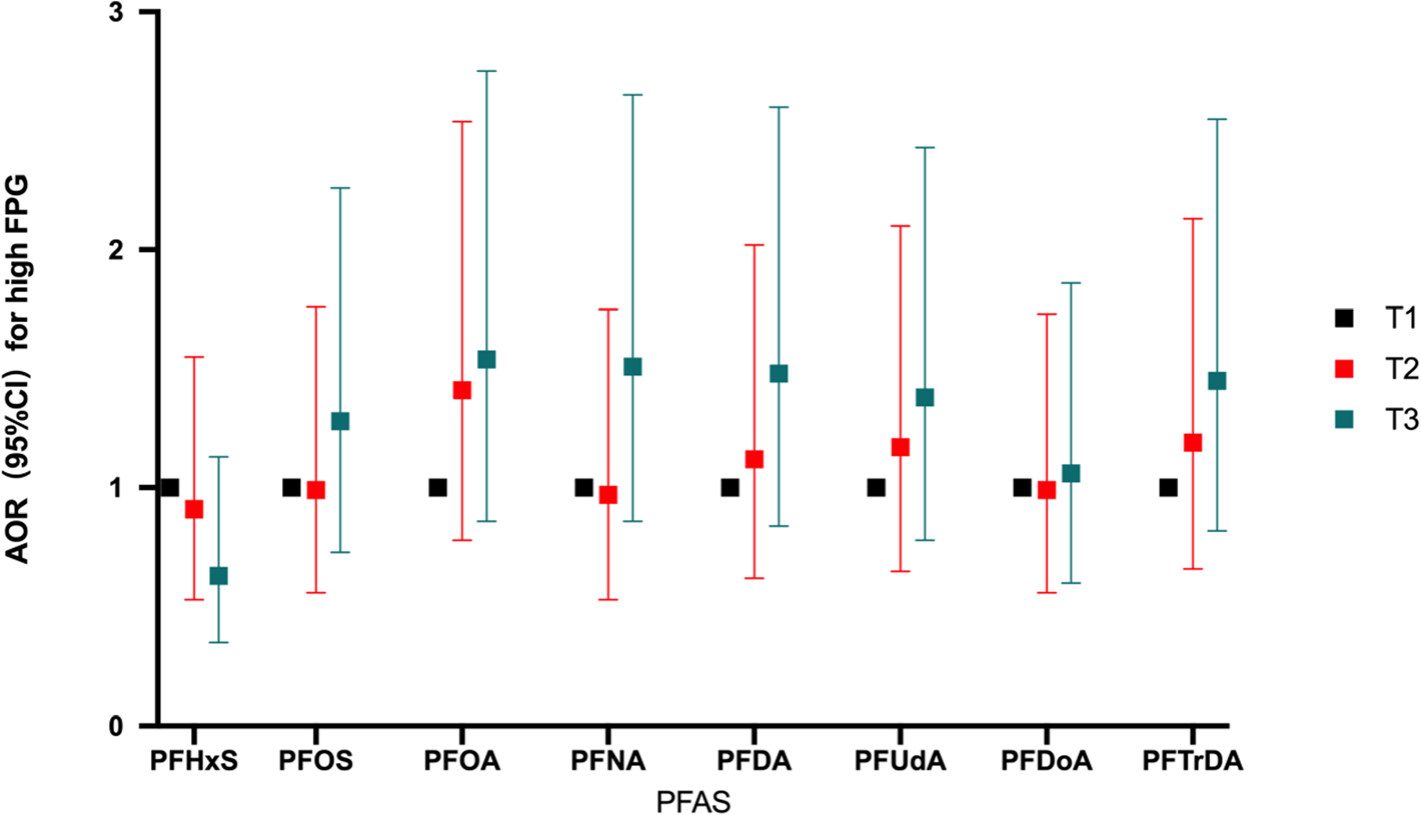
Association between PFAS concentrations (divided by tertiles) and high FPG. Notes: All the *p*-trend values for PFASs with FPG were insignificant

**Fig. 3:**
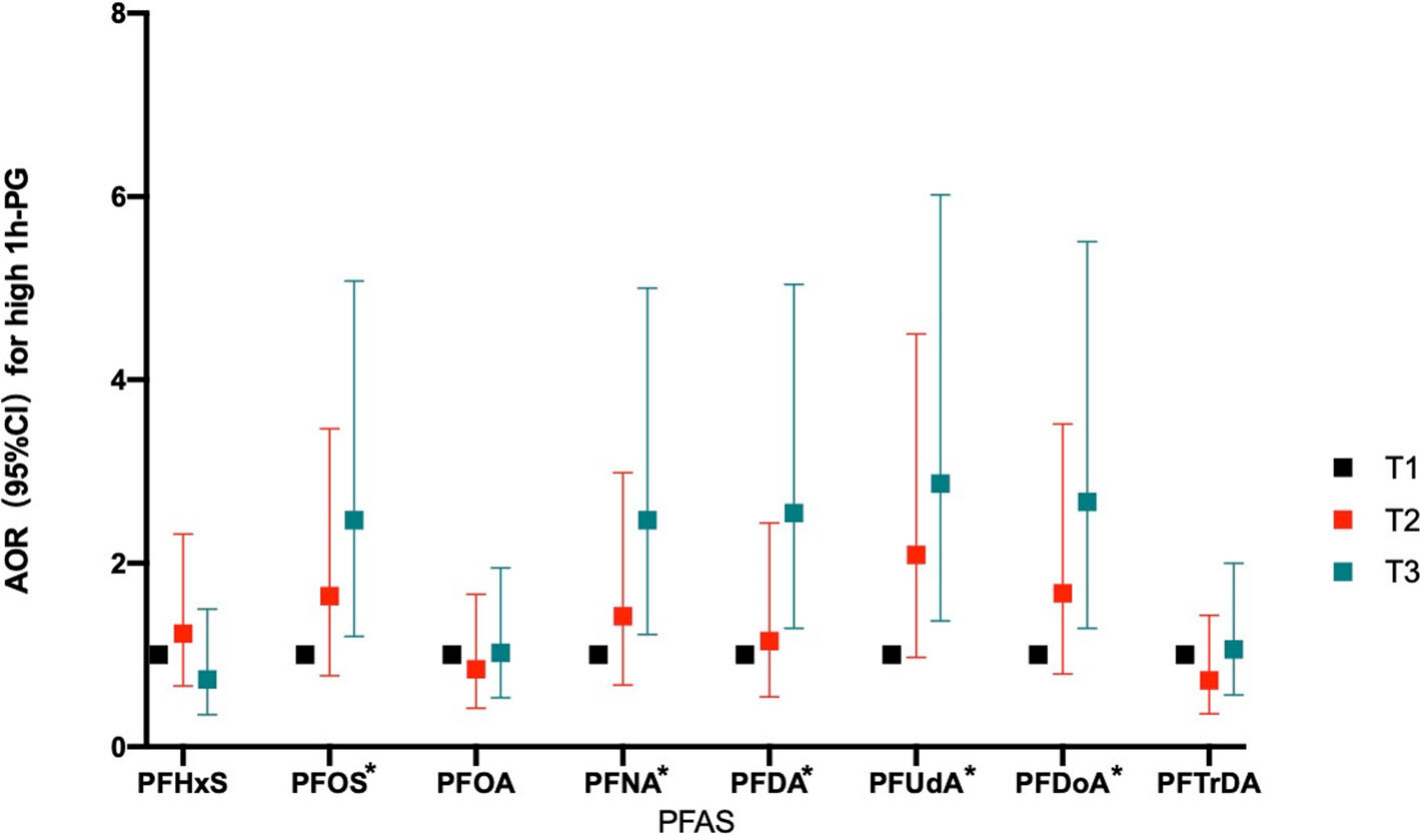
Association between PFAS concentrations (divided by tertiles) and high 1 h-PG. Notes: *p for trend< 0.05

We further examined the associations between the categorized PFAS concentrations and FPG/1 h-PG. Weak associations between the highest tertiles of PFASs and an increased risks of high FPG were observed, but the associations were not statistically significant (Fig. 2). Compared with pregnant women with the lowest tertiles of PFASs, the risk of high 1 h-PG was increased in women with the highest tertiles of PFASs, with statistically significant associations observed for PFOS, PFNA, PFDA, PFUdA, and PFDoA (AOR_PFOS_ = 2.28, 95% CI: 1.09–4.75; AOR_PFNA_ = 2.27, 95% CI: 1.11–4.66; AOR_PFDA_ = 2.37, 95% CI: 1.18–4.73; AOR_PFUdA_ = 2.87, 95% CI: 1.25–5.61; AOR_PFDoA_ = 2.52, 95% CI: 1.21–5.26) (Fig. 3). Linear trends were observed between the tertiles of PFOS, PFNA, PFDA, PFUdA, and PFDoA, and high 1 h-PG (P for trend =0.026, 0.019, 0.011, 0.011, and 0.012, respectively).

We repeated the analysis after excluding women with GDM. The pattern of associations between PFASs and high FPG and 1 h-PG did not change substantially, except that the association between PFDoA and high 1 h-PG was no longer statistically significant (Supplemental Table S3). In addition, the analysis among pregnant women with a BMI of < 25 kg/m^2^ produced similar results (Supplemental Table S4).

In the subgroup analysis for different GW spans, the associations between PFASs and high FPG remained non-significant disregard of the timing of FPG measurements, except that the increased concentrations of PFNA were associated with an increased risk of high FPG at 15–20 GWs (AOR_PFNA_ = 2.54, 95% CI: 1.28–5.07). The pattern of association between PFASs and high 1 h-PG did not substantially change, disregard of measurement time of 1 h-PG, with the exception that the association with high 1 h-PG became non-significant for PFOS, PFUdA, PFDoA (at 20–23 GWs), and PFOS, PFNA, PFDA and PFUdA (at 24–28 GWs), largely owing to the reduced sample size (Supplemental Table S5).

## Discussion

In this prospective cohort study, PFAS exposures in pregnant women were found to be associated with high 1 h-PG, but not FPG, and the association persisted for pregnant women without GDM or with BMI < 25 kg/m^2^.

Many studies have demonstrated that PFASs were associated with impaired glucose homeostasis and an increased risk of diabetes in the general population [17–19]. However, in pregnant women, the associations between PFASs and glucose homeostasis have not been well investigated. Wang et al’s study showed that several PFAS compounds were associated with increased postpartum FPG, including perfluoro-1-metylheptylsulfonat (1 m-PFOS), perfluoro-3/4-metylheptylsulfonat (3 m + 4 m-PFOS), perfluoro-5-metylheptylsulfonat (5 m-PFOS), and PFHxS [26]. The Longitudinal Investigation of Fertility and the Environment (LIFE) study reported that each SD increment in PFOA concentrations was associated with a 1.87-fold increase in GDM risk [21]. In the Odense Child Cohort study, in metabolically vulnerable pregnant women (i.e., BMI ≥27 kg/m^2^, family history of diabetes mellitus, previous GDM, multiple pregnancy, or delivery of a macrosomic child), PFHxS and PFNA concentrations were associated with impaired glycemic status, however, no associations were found in women with low GDM risk [20]. It’s a pity that the absence of information on history of family diabetes and subjects with previous GDM limited our ability of examining the association in subjects with high risk. Higher concentrations of PFASs in our study may partially contribute to the differences with other studies. In our study, concentrations of most PFASs were much higher than those in the Odense Child Cohort [20], the LIFE Study [21] and Wang et al’ study [26] except that PFOS is higher in the LIFE Study compared to the current study. The differences in concentrations, as well as outcome indices of impaired glucose homeostasis, timing of measurement and population included make the comparison between these studies difficult; nevertheless, the potential for PFAS exposure to disturb glucose homeostasis has been supported in most studies.

Although the underlying mechanism linking PFASs to glucose homeostasis is not yet clear, it has been suggested that inhibition of phosphorylation of protein kinase B (Akt) and the activation of peroxisome proliferator activated receptors (PPARs) may play a role [10, 27, 28]. Studies using animal models and HepG2 cells have indicated that PFAS compounds reduce the expression of the phosphatase and tensin homolog protein and affect the Akt signaling pathway [10, 29]. The inhibition of Akt, a key mediator of cellular insulin sensitivity, may stimulate gluconeogenesis and hepatic insulin resistance [29]. Both PFOA and PFOS have been certified to affect glucose metabolism by AKT signaling pathway. However, the other PFASs were not investigated in these studies [10, 29]. In addition, studies have demonstrated that PFASs can bind to and activate the PPAR α and γ receptors [27]. PPAR, a nuclear transcription receptor, is known to play essential roles in the regulation of gene expression, glucose homeostasis, fatty acid metabolism, and inflammation [30]. Therefore, PFAS-activated PPAR could disturb glucose homeostasis by influencing insulin resistance [28] and insulin secretion [31]. PFOA have the highest potential of PPARα activation than the other PFASs with a shorter carbon chain length (including PFHxS, PFNA, PFDA and PFDoA) [32]. Moreover, PFAS exposure may interfere with secretion and function of glucocorticoids and thyroid hormones via hypothalamic–pituitary–adrenal axis and hypothalamic–pituitary–thyroid axis, which may further disturb glucose metabolism [11, 33, 34]. The physiological effects of PFASs on glycemic homeostasis may depend on the potency and concentration of individual PFASs [28, 32].

The strengths of the present study were the prospective nature of the study design, the large sample size, and the measurement of a wide range of PFAS compounds. However, the potential limitations of the study should be considered. First, a considerable proportion of subjects was lost to follow-up, which may have led to selection bias. However, the characteristics of the included subjects were similar to those excluded in terms of age, education, pre-pregnancy BMI, and household income, and thus a substantial selection bias was not expected. Second, the follow-up period from the measurement of PFAS exposure to the endpoints (FPG and 1 h-PG) was short, but the single-point measurement of PFAS concentration may reflect PFAS exposure long before the date of blood collection owing to their long half-life. Third, the relationships between PFASs and blood glucose measures may have been confounded by unmeasured confounders, such as family diabetes history and dietary habits; this should be examined in future studies. In addition, information on maternal active smoking was not collected, since the proportion of active smoking was quite low in Chinese women [35]. For example, only 0.4% of pregnant women have been exposed to active smoking during pregnancy in a Shanghai Birth Cohort [36]. Thus, the current result is not expected to be severely biased by the un-adjustment of active smoking. Fourth, not all the subjects had information on 1 h-PG after the 50-g OGTT, which may have led to missed cases of GDM and affect the association between PFASs and outcome indices in the sensitivity analysis of pregnant women with GDM. However, the absence of GDM cases, if any, would have attenuated the observed association. Fifth, data for FPG (12–20 GWs) or 1 h-PG (20–28 GWs) were collected over a long time span, and thus the associations between PFASs and FPG and 1 h-PG may have been confounded by the gestational week. However, we performed subgroup analyses using different GWs spans at glucose measurement, and found that the results did not change significantly.

## Conclusion

Exposure to certain PFASs (i.e., PFOS, PFNA, PFDA, PFUdA, and PFDoA) was associated with an increased risk of high 1 h-PG among pregnant women. Further studies are needed to clarify the effect of PFASs on gestational glycemic homeostasis and the underlying mechanism.

## Supplementary information

**Additional file 1: Table S1.** The distribution of gestational week at glucose measurement for the included pregnant women. **Table S2.** Association between PFAS concentrations (ln-transformed) and high FPG and 1 h-PG using multiple linear regression. **Table S3.** Association between PFAS concentrations (ln-transformed) and high FPG and 1 h-PG in pregnant women without GDM. **Table S4.** Association between PFAS concentrations (ln-transformed) and high FPG and 1 h-PG in pregnant women with BMI < 25 kg/m^2^. **Table S5.** Subgroup analysis of the association between PFAS concentrations (ln-transformed) and high FPG and 1 h-PG in pregnant women by gestational age. **Figure S1.** Assumed directed acyclic graph for PFASs and plasma glucose.

## Data Availability

The datasets used during the current study are available from the corresponding author on reasonable request.

## Abbreviations

PFASs: Perfluoroalkyl and polyfluoroalkyl substances
FPG: Fasting plasma glucose
1 h-PG: One-hour plasma glucose
GWs: Gestational weeks
PFHxS: Perfluorohexane sulfonate
PFOS: Perfluorooctane sulfonate
PFOA: Perfluorooctanoic acid
PFNA: Perfluorononanoic acid
PFDA: Perfluorodecanoic acid
PFUdA: Perfluoroundecanoic acid
PFDoA: Perfluorododecanoic acid
PFTrDA: Perfluorotridecanoic acid
ORs: Odds ratios
CIs: Confidence intervals
GDM: Gestational diabetes mellitus
OGTT: Oral glucose tolerance test
S-MBCS: Shanghai-Minhang Birth Cohort Study
LOD: Limit of detection
SD: Standard deviations
HOMA-IR: Homeostasis model of assessment for insulin resistance
Akt: Protein kinase B
PPARs: Peroxisome proliferator activated receptors
