# Supplementary material for: Concentrations of perfluoroalkyl and polyfluoroalkyl substances and blood glucose in pregnant women
Source: Environ Health. 2020 Aug 17;19:88. doi: 10.1186/s12940-020-00640-8 (PMC7433207; doi:10.1186/s12940-020-00640-8)
Supplement: Supplementary file 1 — Additional file 1: Table S1. The distribution of gestational week at glucose measurement for the included pregnant women. Table S2. Association between PFAS concentrations (ln-transformed) and high FPG and 1 h-PG using multiple linear regression. Table S3. Association between PFAS concentrations (ln-transformed) and high FPG and 1 h-PG in pregnant women without GDM. Table S4. Association between PFAS concentrations (ln-transformed) and high FPG and 1 h-PG in pregnant women with BMI < 25 kg/m2. Table S5. Subgroup analysis of the association between PFAS concentrations (ln-transformed) and high FPG and 1 h-PG in pregnant women by gestational age. Figure S1. Assumed directed acyclic graph for PFASs and plasma glucose. [file 12940_2020_640_MOESM1_ESM.docx]

**Table S1** The distribution of gestational week at glucose measurement for the included pregnant women

| FPG(N=856) | |  | | | 1h-PG(N=705) | |
| --- | --- | --- | --- | --- | --- | --- |
| Gestational Week | n(%) | |  | Gestational Week | | n( %) |
| 12 | 32(3.74) | |  | 20 | | 12(1.70) |
| 13 | 59(6.89) | |  | 21 | | 39(5.53) |
| 14 | 269(31.43) | |  | 22 | | 100(14.18) |
| 15 | 243(28.39) | |  | 23 | | 208(29.50) |
| 16 | 165(19.28) | |  | 24 | | 226(32.06) |
| 17 | 57(6.66) | |  | 25 | | 89(12.62) |
| 18 | 18(2.10) | |  | 26 | | 23(3.26) |
| 19 | 13(1.52) | |  | 27 | | 8(1.13) |

FPG: fasting plasma glucose; 1h-PG: 1h-plasma glucose after a 50-g oral glucose tolerance test.

**Table S2** Association between PFAS concentrations (ln-transformed) and high FPG and 1h-PG using multiple linear regression

| In-PFAS(ng/ml) | FPG(N=856) | |  | 1h-PG(N=705) | |
| --- | --- | --- | --- | --- | --- |
|  | B (95% CI) | p |  | B (95% CI) | p |
| PFHxS | 0.03(-0.05, 0.11) | 0.456 |  | -0.18(-0.45, 0.09) | 0.195 |
| PFOS | 0.04(-0.02, 0.09) | 0.240 |  | 0.31(0.11, 0.50) | 0.003 |
| PFOA | 0.07(-0.01, 0.14) | 0.097 |  | 0.27(0.03, 0.52) | 0.031 |
| PFNA | 0.09(0.02, 0.16) | 0.008 |  | 0.44(0.22, 0.66) | <0.001 |
| PFDA | 0.03(-0.02, 0.08) | 0.220 |  | 0.22(0.05, 0.39) | 0.011 |
| PFUdA | 0.00(-0.05, 0.04) | 0.929 |  | 0.25(0.10, 0.42) | 0.002 |
| PFDoA | 0.01(-0.02, 0.04) | 0.635 |  | 0.07(-0.04, 0.17) | 0.230 |
| PFTrDA | 0.00(-0.03, 0.03) | 0.909 |  | 0.10(-0.01, 0.21) | 0.071 |

B: regression coefficient; CI: confidence interval; FPG: fasting plasma glucose; 1h-PG: 1h-plasma glucose after a 50-g oral glucose tolerance test.

Models were adjusted for maternal age at enrollment (years), pre-pregnancy BMI (kg/m^2^), per capita household income, education level, passive smoking, pregnancy complication, history of abortion and stillbirth, and parity.

**Table S3** Association between PFAS concentrations (ln-transformed) and high FPG and 1h-PG in pregnant women without GDM

| In-PFAS(ng/ml) | FPG(N=844) | |  | 1h-PG(N=693) | |
| --- | --- | --- | --- | --- | --- |
|  | COR (95% CI) | AOR (95% CI) |  | COR (95% CI) | AOR (95% CI) |
| PFHxS | 1.02(0.62, 1.68) | 0.90(0.51, 1.57) |  | 0.96(0.52, 1.78) | 0.92(0.45, 1.90) |
| PFOS | 1.19(0.81, 1.76) | 1.21(0.80, 1.84) |  | 1.78(1.12, 2.82) | 1.92(1.15, 3.20) |
| PFOA | 1.35(0.82, 2.24) | 1.31(0.76, 2.26) |  | 0.89(0.51, 1.58) | 1.29(0.66, 2.49) |
| PFNA | 1.42(0.92, 2.18) | 1.43(0.89, 2.29) |  | 1.54(0.92, 2.56) | 2.19(1.23, 3.89) |
| PFDA | 1.18(0.85, 1.64) | 1.16(0.81, 1.67) |  | 1.41(0.96, 2.07) | 1.61(1.05, 2.49) |
| PFUdA | 0.95(0.70, 1.29) | 0.93(0.67, 1.28) |  | 1.47(1.00, 2.16) | 1.80(1.15, 2.82) |
| PFDoA | 1.09(0.88, 1.36) | 1.03(0.82, 1.30) |  | 1.27(0.97, 1.67) | 1.38(1.01, 1.88) |
| PFTrDA | 1.02(0.82, 1.26) | 1.09(0.86, 1.37) |  | 1.09(0.84, 1.41) | 1.18(0.88, 1.58) |

COR: crude odds ratio; AOR: adjusted odds ratio; CI: confidence interval; FPG: fasting plasma glucose; 1h-PG: 1h-plasma glucose after a 50-g oral glucose tolerance test; GDM: gestational diabetes mellitus.

Models were adjusted for maternal age at enrollment (years), pre-pregnancy BMI (kg/m^2^), per capita household income, education level, passive smoking pregnancy complication, history of abortion and stillbirth, and parity.

**Table S4** Association between PFAS concentrations (ln-transformed) and high FPG and 1h-PG in pregnant women with BMI<25 kg/m^2^

| In**-**PFAS(ng/ml) | FPG(N=819) | |  | 1h-PG(N=683) | |
| --- | --- | --- | --- | --- | --- |
|  | COR (95% CI) | AOR (95% CI) |  | COR (95% CI) | AOR (95% CI) |
| PFHxS | 0.96(0.58, 1.59) | 0.88(0.50, 1.55) |  | 0.87(0.48, 1.59) | 0.94(0.46, 1.92) |
| PFOS | 1.28(0.87, 1.88) | 1.32(0.86, 2.02) |  | 1.76(1.13, 2.76) | 2.01(1.21, 3.33) |
| PFOA | 1.42(0.86, 2.36) | 1.35(0.78, 2.34) |  | 1.13(0.64, 1.99) | 1.46(0.76, 2.80) |
| PFNA | 1.61(1.04, 2.48) | 1.65(1.02, 2.66) |  | 1.83(1.11, 3.00) | 2.31(1.29, 4.12) |
| PFDA | 1.28(0.92, 1.77) | 1.29(0.89, 1.86) |  | 1.58(1.08, 2.30) | 1.71(1.11, 2.65) |
| PFUdA | 0.98(0.72, 1.34) | 0.98(0.70, 1.36) |  | 1.53(1.05, 2.23) | 1.82(1.17, 2.83) |
| PFDoA | 1.15(0.93, 1.44) | 1.12(0.89, 1.42) |  | 1.20(0.93, 1.56) | 1.30(0.97, 1.76) |
| PFTrDA | 1.06(0.86, 1.31) | 1.14(0.90, 1.45) |  | 1.07(0.84, 1.37) | 1.11(0.84, 1.47) |

COR: crude odds ratio; AOR: adjusted odds ratio; CI: confidence interval; FPG: fasting plasma glucose; 1h-PG: 1h-plasma glucose after a 50-g oral glucose tolerance test.

Models were adjusted for maternal age at enrollment (years), pre-pregnancy BMI (kg/m^2^), per capita household income, education level, passive smoking, pregnancy complication, history of abortion and stillbirth, and parity.

**Table S5** Subgroup analysis of the association between PFAS concentrations (ln-transformed) and high FPG and 1h-PG in pregnant women by gestational age

| In-PFAS | AOR (95% CI) for high FPG | |  | AOR (95% CI) for high 1h-PG | |
| --- | --- | --- | --- | --- | --- |
|  | At 12-14 GWs  N=360 | At 15-20GWs  N=496 |  | At 20-23 GWs  N=359 | At 24-28 GWs  N=346 |
| PFHxS | 0.62(0.24, 1.60) | 1.05(0.51, 2.15) |  | 1.12(0.42, 2.98) | 0.76(0.27, 2.01) |
| PFOS | 1.12(0.62, 2.04) | 1.43(0.80, 2.56) |  | 1.97(0.97, 3.99) | 1.90(0.93, 3.87) |
| PFOA | 0.95(0.44, 2.06) | 1. 82(0.86, 3.87) |  | 1.02(0.43, 2.41) | 2.06(0.83, 5.06) |
| PFNA | 0.96(0.50, 1.84) | 2.54(1.28, 5.07) |  | 2.69(1.20, 6.04) | 1.87(0.84, 4.12) |
| PFDA | 0.98(0.58, 1.65) | 1.50(0.91, 2.46) |  | 1.99(1.05, 3.79) | 1.43(0.81, 2.50) |
| PFUdA | 0.81(0.53, 1.26) | 1.16(0.72, 1.88) |  | 1.80(0.95, 3.40) | 1.69(0.94, 3.02) |
| PFDoA | 1.12(0.78, 1.59) | 1.06(0.78, 1.45) |  | 1.17(0.78, 1.76) | 1.58(1.01, 2.49) |
| PFTrDA | 1.02(0.72, 1.44) | 1.20(0.88, 1.63) |  | 1.18(0.81, 1.71) | 1.04(0.68, 1.60) |

AOR: adjusted odds ratio; CI: confidence interval; FPG: fasting plasma glucose; 1h-PG: 1h-plasma glucose after a 50-g oral glucose tolerance test; GWs: gestational weeks.

Models were adjusted for maternal age at enrollment (years), pre-pregnancy BMI (kg/m^2^), per capita household income, education level, passive smoking, pregnancy complication, history of abortion and stillbirth, and parity.


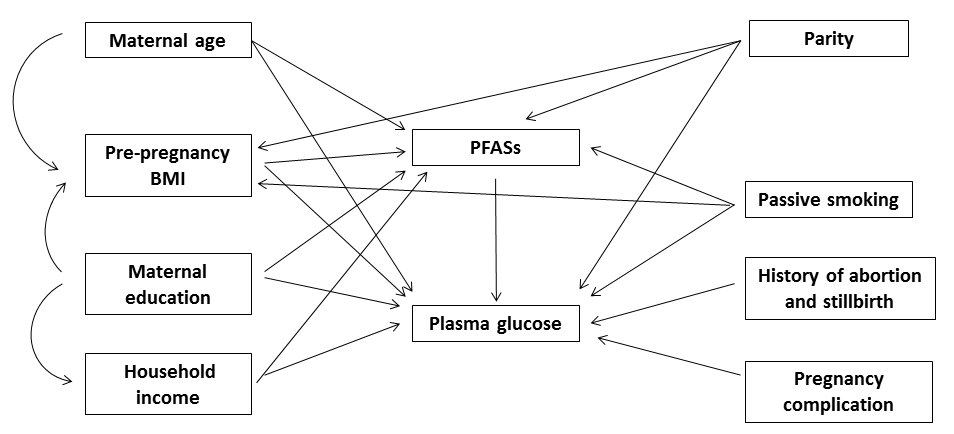


Figure S1 Assumed directed acyclic graph for PFASs and plasma glucose
